# Supporting breast and cervical cancer screening follow-up: qualitative health plan interviews

**DOI:** 10.1186/s12913-026-14641-9

**Published:** 2026-05-02

**Authors:** Brenna Lin, Kathleen Gruschow, Alana Burke, Xiaofei Zhou, Thomas B. Richards, Sepheen C. Byron

**Affiliations:** 1 Quality Measurement and Research Group, National Committee for Quality Assurance, 1100 13th St NW 3rd floor, Washington, DC 20005 USA; 2 Division of Cancer Prevention and Control, National Center for Chronic Disease Prevention and Health Promotion, Centers for Disease Control and Prevention, Atlanta, Georgia

**Keywords:** Health care quality, Cancer screening, Cancer follow-up

## Abstract

**Background:**

Breast and cervical cancer represent a significant disease burden. Health care quality measures captured through electronic clinical data systems could be used to assess follow-up of abnormal breast and cervical cancer screening results. The National Committee for Quality Assurance (NCQA) wanted to explore the feasibility of using health plan electronic clinical data systems to develop quality measures for follow-up of abnormal breast and cervical cancer screening test results.

**Methods:**

NCQA interviewed quality improvement leaders from ten health plans, recruited through a purposive sample representing varying sizes, geographic regions, and commercial, Medicare, and Medicaid product lines.

**Results:**

Health plan leaders identified several barriers, including a lack of standardized data integration as well as opportunities such as supporting provider and health system data aggregation and other support.

**Conclusions:**

Findings indicate that improved data governance, particularly around data standardization and information technology infrastructure integration, would increase the feasibility of monitoring follow-up after abnormal breast and cervical cancer screening results.

**Table:** 

Text box 1. Contributions to the literature
• This paper summarizes perspectives from health plan representatives on barriers and opportunities for breast and cervical cancer screening follow-up.
• Health plan representatives highlighted some challenges in obtaining and structuring data needed for tracking follow-up.
• We suggest considerations for data governance to support improving quality of care for patients with abnormal breast and cervical cancer screening results.

## Background

Breast and cervical cancers represent a significant disease burden. In 2020, there were 4272 reported deaths related to cervical cancer and 42,273 deaths related to breast cancer in the United States [1, 2]. While there has been some success in disease management due to increased attention on screening programs, outcome improvement relies on proper follow-up of abnormal test results [3]. Currently, however, follow-up of abnormal results is less than ideal. For example, one study in the United States found follow-up for abnormal Papanicolaou (Pap) results ranged from 28% to 53% [4]. Another study, a systematic literature review of publications primarily in the United States, found that up to 30% of women did not receive recommended immediate follow-up for high-risk mammograms [5]. Various patient, physician, and health system factors contribute to these follow-up rates [5, 6]. Individual patient factors can include fear of pain or embarrassment, childcare or transportation issues, and insurance status; physician-level factors can include inadequate patient communication, the effectiveness of clinician reminders and inability to identify patients’ result data; health system factors can include requiring a referral and the timeliness and availability of those referrals, available appointment times for mammograms, and having other available resources such as patient navigators or case management [5, 6].

Failure to complete timely diagnostic testing undermines the significance of screening and can result in poorer health outcomes [7]. A review conducted by the Population-based Research Optimizing Screening through Personalized Regimens (PROSPR) consortium, which includes research centers across healthcare systems in the United States, found that delays between a positive cancer screening result and diagnostic follow-up are associated with increased risks of cancer progression, advanced-stage diagnosis, and mortality. The study focused on breast, cervical, colorectal, and lung cancers – which all have established screening guidelines – and emphasized the importance of timely follow-up to improve outcomes and reduce disparities [7]. Another consortium study also estimated that the probability of abnormal findings in cervical cancer screenings is around 4%-5% and that follow-up within three months is about 45%; for breast cancer screenings, approximately 11% of screenings result in abnormal findings, while follow-up within three months is much higher at 96% [3, 8]. It is also important to consider that some populations in the United States experience greater delays in care than others. Compared to non-Hispanic White women, African American, Hispanic, and Asian women, as well as women with underlying health conditions tend to experience longer delays in follow-up care for abnormal mammograms [9–11]. This delayed time to follow-up may result in advanced disease stages, ultimately playing a role in the decreased survival rates among women in minority groups and women in communities underserved by adequate access to medical care [10]. Similarly, cervical cancer predominantly affects women in often marginalized groups; [12] such groups include racial and ethnic minorities, sexual and gender minorities, certain religious groups, and people residing in rural areas, with limited proficiency in English, and with various medical conditions [13]. 

In the United States, a health plan provides insurance medical coverage to their members. Health plans finance health coverage and are held responsible for managing patients’ care [14]. Healthcare quality measures can assess health plan performance, drive improvements in outcomes, and provide consumers, health plans and policymakers with information on how well health plans perform annually on critical dimensions of care [15]. Quality metrics do this through the translation of clinical guidelines into data specifications that are measurable across entities. A health plan can use patient healthcare data from claims and electronic clinical data, including electronic health records (EHRs) to assess healthcare quality [16]. For example, in addition to whether a patient received a cancer screening test, clinical data found in medical records can provide information on the test results, and whether abnormal results received appropriate and timely follow-up. In 2024, quality measures existed for breast cancer screening and cervical cancer screening within Healthcare Effectiveness Data and Information Set (HEDIS^®^), but no measures existed tracking the follow-up of abnormal tests. In the United States, health plans often use HEDIS measures to identify patients that are out of compliance with their recommended care. These care gaps flag health plan care teams and providers that screening or other care is due. Importantly, identifying patients in earlier stages of breast and cervical cancer can reduce treatment costs for both the patient and the health plan [17, 18]. The absence of quality measures associated with abnormal screening combined with reported challenges health plans have faced in collecting clinical data may result in health plans not readily storing and tracking screening test results across their members [19]. The development of quality measures focused on follow-up can help streamline and incentivize health plans and system operations to identify individuals eligible for follow-up care.

Recognizing the increasing use of electronic Clinical Quality Measures (eCQMs) in US healthcare [16], the National Committee on Quality Assurance (NCQA) has started to design computable quality measures for use with electronic clinical data systems in the HEDIS measure set [20]. In addition to existing quality measures that assess the proportion of eligible persons that received a screening test, NCQA explored the feasibility of using health plan electronic clinical data systems to develop quality measures for follow-up of abnormal breast and cervical cancer screening test results.

In this qualitative study, we sought to understand barriers, opportunities, and potential feasibility of health plans use of their electronic clinical data systems to report quality performance measures for follow-up of abnormal breast and cervical cancer screening results. Abnormal breast cancer screening results include mammograms that are American College of Radiology Breast Imaging-Reporting and Data System (BI-RADS) 0 (need for additional imaging or prior mammograms), 4 (suspicious), or 5 (highly suggestive of malignancy) [21]. Abnormal results for cervical cancer screening include Pap-stained cervical cytology tests that reveal pre-cancerous or cancerous processes in the cervix, or the identification of high-risk human papillomavirus (HPV) genotypes associated with a higher risk of cervical cancer [22].

## Methods

NCQA used the NCQA Quality Compass data to identify and recruit a purposive sample of diverse plans regarding enrollment size, location, and product line reporting a mix of performance rates on the Breast Cancer Screening and Cervical Cancer Screening HEDIS measures. We identified health plans with rates that were high and low, as well as with rates that improved or declined over the past 3 years. High and low performance was determined by comparing the health plan’s performance to the national means, by product line, between years 2019, 2020, and 2021. Forty health plans were identified for outreach as potential interviewees, with the goal of conducting 9–12 total interviews [23]. We identified points of contact through NCQA’s customer relationship management software and sent email invitations to the selected health plans with the project background and asked potential participants to identify the most appropriate people to interview. We sent out invites to 40 health plan contacts. Participants who did not respond to our email after two follow-ups were dropped from consideration as participants. The first 10 health plans that responded were interviewed. We developed an open-ended, semi-structured interview guide. An initial guide was developed by the lead author and shared with coauthors that included topics such as successes, challenges and data infrastructure used to support successful breast and cervical cancer screening follow-up. Coauthors’ feedback was incorporated into the final version. Questions in the guide were used flexibly, adapted, or elaborated on according to the discussion to prompt dialogue and explore themes further. We audio-recorded and transcribed the interviews with all study participants’ informed consent. The lead author conducted all interviews, while coauthors participated with follow-up or clarifying questions. A majority of the authors are employed by NCQA. The team has experience conducting both in-person and virtual interviews with group sizes ranging from one to ten.

The team reviewed the transcripts and conducted a thematic analysis. We coded and analyzed the transcripts using Braun and Clarke’s six-phase thematic analysis [24]. This process of thematic analysis is driven by identifying meaning of patterns by (1) Familiarizing oneself with the data, (2) Generating codes, (3) Constructing themes, (4) Reviewing potential themes, (5) Defining and naming themes, and (6) Producing the report [24]. Patterns were identified inductively – first, from the interviews themselves, initial codes were identified. Codes were then re-evaluated during the analysis of the interview transcripts and organized into broad themes by two of the authors. The themes were then reevaluated in consultation with authors. No coding software was used. Participants did not receive any form of reimbursement for their participation in this study. Due to the practical and administrative nature of this project, it was determined to be a clinical healthcare quality improvement study and does not meet the Health and Human Services definition of “research” as defined in 45 CFR 46.102(d) [25]. Thus, this study was exempt from review by an Institutional Review Board.

## Results

We conducted qualitative interviews with senior quality improvement and reporting staff at ten health plans in April 2023 via video calls that lasted 1–1.5 h (Table 1). Compared to the national health plan average enrollment, plan size was small (less than 25% of the average) in three plans, medium (25%-75% of the average) in four plans, and large (greater than 75% of the average) in three plans. Plan catchment areas were in the Northeast (one plan), Midwest (two plans), South (three plans), West (two plans), and multiple regions (two plans) of the United States. Six plans were Medicaid Health Maintenance Organizations (HMOs), one was a Medicare Preferred Provider Organization (PPO), two were Commercial PPOs, and one was a Commercial HMO and PPO. Although all plans represented reported the existing HEDIS quality measures for breast and cervical cancer screening, most interviewees stated that their organizations did not track abnormal results after screening. Interviewees that did track abnormal results mentioned that they did not have this information consistently across their population. 

**Table 1:** Characteristics of participants and their health plans

Size^1^	Region	Plan Types^2^	Interviewee Position Titles
Small	Midwest	Medicaid HMO	Senior Manager of Quality Improvement and Compliance
Small	West	Medicaid HMO	Senior HEDIS Planning Analyst; Director of Quality Metrics; Internal Medicine Physician; Health Plan Director; Diagnostic Imaging Manager; Director for Quality Integration; Assistant Administrator for Quality; Director of Analytics; Senior Manager for Population Health
Small	South	Commercial HMO and PPO	Director of HEDIS Operations and Compliance
Medium	Multiple	Medicare PPO	Director of Quality Strategy on National STARS and HEDIS Team; Associate Director on Quality Optimization and Insights Team
Medium	South	Medicaid HMO	Analytics Manager; Director of Quality and Clinical Analytics
Medium	Northeast	Medicaid HMO	Program Manager; Program Manager under Chief Medical Officer; Senior Manager for Care Management Team; Director of Care Management; Director of Corporate Quality
Medium	West	Commercial PPO	Senior Director- Clinical Quality and Risk Adjustment; Quality Submissions Program Manager; Quality Performance Manager
Large	Midwest	Medicaid HMO	Assistant Vice President- Member Engagement & Interventions
Large	Multiple	Commercial PPO	Director of Quality and Clinical Oversight; Population Health Program Manager; Director of Health Plan Administration
Large	South	Medicaid HMO	Director of Clinical Quality; Health Equity Director; Whole Health Director; OB Practice Consultant; Accreditation and Quality Programs Executive Producer; Quality Lead; Director of Medicaid

^1^ Plan size was determined through the distribution of enrollment counts over the past two years. Small health plans had enrollment numbers that fell below the 25th percentile. Medium-size plans had enrollment numbers that ranged between the 25th and 75th percentiles. Large-sized plans had enrollment numbers greater than 75% percentile

^2^ PPO is a preferred provider organization. HMO is a health maintenance organization

Additionally, the plans identified several barriers and opportunities for data governance related to the tracking and management of follow-up for abnormal breast and cervical cancer screening results (see Table 2).

### Barriers

#### Lack of data standardization

Most health plan representatives reported that data on abnormal clinical results varied in completeness and usefulness and that different reporting sources often used different formats, variables, and definitions. A participant shared “I’d say the majority of it comes in kind of unstructured, and then we take it and make as much sense out of it, in our systems, as possible.”[Medium-sized, Northeast Health Plan] Clinical electronic record systems frequently only had abnormal results in an unstructured format, such as PDF reports from the radiology department on a mammogram or from the pathology department about cytology on a Pap test, often difficult to process. The same participant said “if it’s storing something like a PDF….there still is going to need to be that human contact… we’re not to the point where we can just receive a PDF and … recognize, oh, here’s that code set built, and then place it where it needs to go.” [Medium-sized, Northeast Health Plan] Most health plan representatives also shared another issue related to lack of standardization is the lack of industry agreed upon definitions, “what’s even more problematic with the incomplete information is how to capture all the high risk… family history, genetic history, that may not all be clean in the medical record.” [Small-Sized, Western Health Plan] Different eligibility criteria for screening and follow-up also exist, “different systems have different guidelines they like to go by. And there’s some controversy between the different types of physicians (primary care vs. specialists), and their approach that they like to go for…so there’s difficulty in implementing a standard approach right now.” [Medium-sized, Southern Health Plan] A few plans noted that once industry standardized codes are agreed upon, Natural Language Processing (NLP) and other artificial intelligence (AI) algorithms could be used to extract and standardize manually entered clinical results from notes in patient electronic records.

#### Lack of information technology infrastructure integration

All health plan representatives reported struggling to access complete patient healthcare data from EHRs, case management and disease management systems. Many records are still paper based and requests for these records are often time consuming or incomplete. A participant shared that it “might take 7 to 10 business days — if you ever get a response.“[Medium- sized, Multi-region Health Plan] All plans have recognized that increased use of EHR information systems could help improve and speed access. Still, a challenge has been the lack of standardization and integration within and across information systems used by health providers. One health plan representative reported, “We have over 200 EHR systems across all networked providers.” [Medium-sized, Southern Health Plan] All plan representatives also note that even where data infrastructure is integrated, they often needed to do extensive additional work to clean and standardize the data; “it comes in and has to be normalized… so on our end it speaks the same language.” [Large-sized, Multi-Region Health Plan].

### Opportunities

#### Data aggregation

Some health plan representatives noted they are beginning to explore data aggregation solutions, such as establishing data aggregators, connecting with local or state health information exchanges, and working to receive data from larger provider groups. One respondent said they have actively “reached out to more and more of our different provider groups… to establish direct connections.” [Medium-sized, Southern Health Plan] A interviewee from a different health plan said “Today, we have connections with 40 state information exchanges.“[Small-sized, Southern Health Plan] Another respondent also noted, “over the last six months I think we’ve set up four or five different aggregators … and then local state HIEs (Health Information Exchanges) to collect and obtain as much of this information, digitally and directly as possible to make the heavy lift of getting this information a lot easier, safer, and quicker, so we can make better decisions faster.” [Medium-Sized, Northeastern Health Plan] Furthermore one health plan representative described a strategy to overcome data obstacles by “acting as an interoperability hub and bringing in all data to run an analytic engine that can provide more accurate reporting.” [Small-Sized, Western Health Plan].

#### Health system and provider support

A few health plan representatives reported the highest engagement when members received information from the providers, such as when a provider informs patients about benefits vs. the harms of screening. One health plan representative was a breast cancer survivor and shared they did not find it appropriate for health plans to communicate care plans. “I’m a (breast cancer) survivor, and I would have thought it would be really weird if my health plan reached out during that time… during that time, you latch on to your (clinical) team…to help them navigate next steps.” [Large-sized, Midwestern Health Plan].

Similarly, a few other health plan representatives noted avoiding member abrasion, when possible. “We want to make sure that we are not causing member abrasion by saying “Mrs. Smith needs her Pap smear,” but she just got it two months ago.” [Large-sized, Southern Health Plan] A majority of health plans sought ways to support patient-clinician relationships by assisting health system and providers in helping members understand the clinical implications of their results and their medical benefits, identifying gaps and barriers to care, and creating and distributing educational materials. For example, a participant shared that they can inform patients on the “availability of our radiology (department), that sometimes they do have walk-in availability and really support…outreach efforts” [Small-Sized, Western Health Plan] One interviewee said the plans “could offer to work together and collaborate… treating it more like we’re part of the medical team.” [Medium-sized, Southern Health Plan] Another participant shared “primarily, our largest approach is to try to support providers by spreading best practices” [Small-Sized, Midwestern Health Plan] Health plans could also consider developing data flow diagrams and field guides to offer technical support to provider groups or provide primary source verification and audits of incoming data for quality assurance.

## Discussion

Through comprehensive data governance, health care organizations can improve the tracking and management of those eligible for follow-up after abnormal breast or cervical cancer screening results. Health plan interview respondents emphasized the importance of ensuring digital connectivity, security, consistency, and conciseness of data across different data feeds with a goal of gathering accurate, timely, measurable, and actionable data. While interviewees noted many challenges with unstructured data, this is changing due to several national efforts. Health Level (HL7^®^) standards, such as Fast Healthcare Interoperability Resources (FHIR^®^), are being adopted to provide the infrastructure for the exchange, integration, and retrieval of health data. To streamline exchange of data between stakeholders, the Office of National Coordinator for Health Information Technology (ONC) 21st Century Cures Act mandated that providers must implement HL7^®^ FHIR^®^ application programming interfaces [26]. Furthermore, NLP, large language models (LLMs), and other AI models that can extract or interpret information from unstructured free-text data, such as data included in the notes sections of EHR, can improve efficiency and effectiveness of data standardization [27]. Health plan and system decision makers should consider opportunities to improve the accuracy and interoperability of their patient data.

The development and exchange of standardized information is an active and challenging pursuit for many in the health ecosystem. There are many ways in which health plans are uniquely positioned to support standardized data exchange. Health plans could also consider developing data flow diagrams and field guides to offer technical support to provider groups or offer primary source verification and audit incoming data as a form of quality assurance. See Table 2 for a summary of barriers and opportunities.

**Table 2:** Themes and Key Discussion Points: Barriers and Opportunities for Health Plan Follow-up of Abnormal Breast and Cervical Cancer Screening Results

Themes	Key Discussion Points
Barriers	
Lack of data standardization	Abnormal cancer screening results are not coded in a standardized, structured format.
	Information may need to be extracted from PDF reports and clinical notes.
	Historical information (e.g., family history, past high-risk human papillomavirus test and cervical cytology results) may require health provider office visit time to enter data by hand.
	Different medical specialties may differ on definitions of high-risk individuals and the appropriate follow-up management after an abnormal cancer screening test.
Lack of information system integration	Different health providers often have different information technology infrastructures and clinical data systems.
	Patient clinical management may require data from multiple data sources, health providers, information systems, locations, and time periods.
	Data from various sources may not be integrated with, easily available, or usable within the health plan data infrastructure.
Opportunities	
Data aggregation	Health plans could establish integrated interoperability hubs to evaluate and report quality measures.
	Health plans could help identify key variables that need to be coded and available in a standardized, structured format for easy analysis.
Health system and provider support	Health plans could employ learning collaboratives and clinical teams to identify gaps in care and support members.
	Health plans could develop data flow diagrams, provide technical support to provider groups, and conduct quality audits of incoming data.

The development and implementation of eCQMs, such as NCQA’s development of quality measures within HEDIS, can facilitate and incentivize data improvement by offering clear specifications and standardized definitions [19] as well as improve the feasibility of tracking and managing abnormal breast and cervical cancer screening follow-up and ultimately improve health outcomes for these diseases [15]. Better data, shared more freely in a timely manner, can appropriately identify patients for screening and follow-up and making sure they are receiving the appropriate care. For example, cervical cancer screening and follow-up can be conducted either by a primary care physician or a gynecologist. If the two physicians work in different health systems, data may not be shared freely. This can result in the patient losing track of their recent results or result history and possibly falling through care gaps, especially if neither physician has complete patient history. Notably, history of cervical screening results is complex and risk-based – various combinations of cytology and HPV screenings result in different recommended follow-up actions at different time frames [28]. FHIR enabled data would allow providers and health plans to more readily see and document an abnormal results to coordinate care; physicians could follow-up with the patient and prioritize a referral (if needed), while health plans can share benefit and coverage information with the member.

In August 2024, this team presented to NCQA’s voting body on the development of two new electronic clinical data measures related to mammogram assessment and follow-up [29]. The first measure assesses the proportion of mammograms that had a documented assessment within 14 days of the mammogram for members aged 40–74. The second measure assesses the proportion of inconclusive or high-risk assessments that received appropriate follow-up within 90 days of the inconclusive or high-risk assessment for members aged 40 to 74. The two measures released in HEDIS for breast cancer screening follow-up will incentivize standardized data capture and exchange between health providers, systems and health plans for all mammogram results. The follow-up measure will assess timely follow-up for abnormal results. Through management and use of these measures in programs, health plans will be held accountable to high-quality care provided at the right time. The cervical cancer screening follow-up measure was considered not feasible at this time, in part because health plan clinical electronic data systems did not currently capture the specificity required by the Enduring Consensus Cervical Cancer Screening and Management Guidelines, such as details about HPV genetic strains [22]. 

The breast cancer screening follow-up quality measures were released in August of 2025 as first-year measures. NCQA will receive and begin to analyze health plan submitted data in Quality Compass in the summer of 2026. First year measures allow NCQA to capture data from health plans, without producing national benchmarks within Quality Compass (public reporting status). After a review of the data, the team will recommend if the measures stay in first year status or move to public reporting status. As coding standards evolve, NCQA will evaluate opportunities to continue development of a cervical cancer screening follow-up measure.

The study includes at least three limitations. First, the results may not be representative because the study included a relatively small number of interviews, and health plan participants were not randomly selected. Health plan representatives responsive to our request for interview may be more engaged in data quality improvement efforts than those that did not respond to our interview requests. Second, interview respondents were primarily quality improvement staff, and comments from other perspectives might be different (e.g., from other operations staff, different categories of health care providers, or health plan information technology experts). Across plans, those interviewed had different levels of seniority, cross-departmental collaboration, and resources available at their disposal. Third, all health plans interviewed are accredited by NCQA so results may be biased by social desirability.

## Conclusion

To improve patient care, all health care organizations could consider opportunities to standardize data, integrate with larger existing data sources, and use structured interoperable data as well as consider their role in fostering greater data governance. In particular, health plans are in a unique position to incentivize tracking timeliness of appropriate follow-up care after abnormal breast and cervical cancer screening results. Health plans can support patients, health care providers and health systems by serving as an interoperability hub by aggregating and structuring multiple data sources and linking a member’s enrollment across time and spaces. This could enable a health plan to improve data governance and support data integration and sharing among primary care physicians, specialists, and other providers to foster timely follow-up after abnormal breast or cervical cancer screening results.

## Data Availability

The dataset generated and analyzed during the current study is not publicly available due to the highly sensitive nature of interview transcript data. Publication of entire transcripts risk identifying research participants.

## Abbreviations

AI: Artificial Intelligence
BI-RADS: Breast Imagining- Reporting and Data System
eCQMs: Electronic Clinical Quality Measures
EHR: Electronic Health Record
FHIR: Fast Healthcare Interoperability Resources
HEDIS: Healthcare Effectiveness Data and Information Set
HIE: Health Information Exchange
HL7^®^: Health Level
HPV: Human Papillomavirus
LLM: Large Language Model
NCQA: National Committee for Quality Assurance
NLP: Natural Language Processing
ONC: Office of National Coordinator for Health Information Technology
Pap: Papanicolaou
